# q2-longitudinal: Longitudinal and Paired-Sample Analyses of Microbiome Data

**DOI:** 10.1128/mSystems.00219-18

**Published:** 2018-11-20

**Authors:** Nicholas A. Bokulich, Matthew R. Dillon, Yilong Zhang, Jai Ram Rideout, Evan Bolyen, Huilin Li, Paul S. Albert, J. Gregory Caporaso

**Affiliations:** a The Pathogen and Microbiome Institute, Northern Arizona University, Flagstaff, Arizona, USA; b Merck & Co., Inc., Kenilworth, New Jersey, USA; c Departments of Population Health (Biostatistics) and Environmental Medicine, NYU Langone Medical Center, New York, New York, USA; d Biostatistics Branch, Division of Cancer Epidemiology and Genetics, National Cancer Institute, Rockville, Maryland, USA; e Department of Biological Sciences, Northern Arizona University, Flagstaff, Arizona, USA; University of Copenhagen

**Keywords:** bioinformatics, linear mixed effects, longitudinal analysis, microbiome

## Abstract

Studies of host-associated and environmental microbiomes often incorporate longitudinal sampling or paired samples in their experimental design. Longitudinal sampling provides valuable information about temporal trends and subject/population heterogeneity, offering advantages over cross-sectional and pre-post study designs. To support the needs of microbiome researchers performing longitudinal studies, we developed q2-longitudinal, a software plugin for the QIIME 2 microbiome analysis platform (https://qiime2.org). The q2-longitudinal plugin incorporates multiple methods for analysis of longitudinal and paired-sample data, including interactive plotting, linear mixed-effects models, paired differences and distances, microbial interdependence testing, first differencing, longitudinal feature selection, and volatility analyses. The q2-longitudinal package (https://github.com/qiime2/q2-longitudinal) is open-source software released under a 3-clause Berkeley Software Distribution (BSD) license and is freely available, including for commercial use.

**IMPORTANCE** Longitudinal sampling provides valuable information about temporal trends and subject/population heterogeneity. We describe q2-longitudinal, a software plugin for longitudinal analysis of microbiome data sets in QIIME 2. The availability of longitudinal statistics and visualizations in the QIIME 2 framework will make the analysis of longitudinal data more accessible to microbiome researchers.

## Abstract (precis)

Longitudinal sampling provides valuable information about temporal trends and subject/population heterogeneity. We describe q2-longitudinal, a software plugin for longitudinal analysis of microbiome data sets in QIIME 2. The availability of longitudinal statistics and visualizations in the QIIME 2 framework will make the analysis of longitudinal data more accessible to microbiome researchers.

## INTRODUCTION

Time is an important component in many microbiome studies. Sampling microbial communities repeatedly over time provides information on their development (1), stability (2–4), or response to and recovery from a treatment or disturbance (5–7). The frequency and scale of longitudinal sampling can range from pre-post studies, in which individual subjects are sampled before and after treatment (8), to long-term observation studies lasting months or years. Such studies benefit from the use of dynamic analytical methods, which evaluate trends over time in relation to one or more variables, paired methods, which evaluate the magnitude of change within individual subjects, and random-effects models, which account for the variation inherent to complex biological systems (9).

To facilitate routine application of appropriate longitudinal methods in microbiome studies, we developed q2-longitudinal (https://github.com/qiime2/q2-longitudinal), a suite of bioinformatics tools for paired and longitudinal analyses. This software package is a plugin for the microbiome bioinformatics platform QIIME 2 (https://qiime2.org/) and, thus, adopts the software architecture, multiple-user interfaces (including a graphical user interface), provenance tracking, and other user benefits offered by QIIME 2. This plugin includes several novel longitudinal analysis and plotting methods, described below. In addition, many of the analyses provided in q2-longitudinal wrap preexisting tools, streamlining their use and reducing the burden for users to install, run, and interpret. Other analyses adapt standard statistical approaches for microbiome data (nonparametric tests are used by default, but parametric equivalents are supported for some plugin actions). All analyses are provided as easy-to-use extensively unit-tested pipelines, outputting interactive, publication-ready plots and tables that are generated by q2-longitudinal, which adds additional value relative to using the underlying tools directly.

## RESULTS AND DISCUSSION

**FIG 1:**
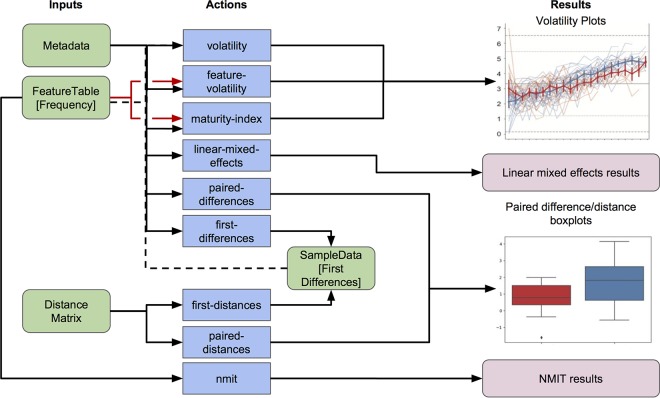
Schematic overview of q2-longitudinal. Green boxes indicate QIIME 2 artifact files, labeled by the file type/format. Blue boxes indicate actions (the various functions available in q2-longitudinal), labeled by the function name. Lines indicate required inputs and outputs; dotted lines indicate optional inputs. All actions require sample metadata files, and feature tables (sample by observation matrices, e.g., of operational taxonomic units [OTUs], taxa, or sequence variant data) are optional inputs to a number of actions but required by the feature-volatility and “maturity-index” pipelines (red arrows for emphasis). Only some actions shown in this schematic are described in this work; see https://github.com/qiime2/q2-longitudinal or https://qiime2.org for more details on all actions available in q2-longitudinal. NMIT, Non-parametric Microbial Interdependence Test.

The q2-longitudinal plugin is designed to facilitate streamlined analysis and visualization of longitudinal data sets, offering a range of tools for longitudinal and paired-sample analysis, including the nonparametric microbial interdependence test (10) and linear mixed-effects (LME) models (9) (Fig. 1). The implementation of interactive visualizations (as volatility plots) and supervised regression pipelines for identifying longitudinally volatile features in particular are novel offerings of this plugin, allowing users to quickly and interactively explore longitudinal data.

### Volatility charts.

The temporal stability or volatility of a metric between individual subjects or groups of subjects can be an important measurement, indicating periods of disruption, disease, or abnormal events. Microbial volatility, the variance in microbial abundance, diversity, or other metrics over time, can be a marker of ecosystem disturbance or disease (4, 11, 12) and hence provides another important metric for comparison between experimental groups. We can visualize these fluctuations using control charts, which show how a variable changes over time in individuals or groups. These charts display “control limits” 3 standard deviations above and below the mean and “warning limits” 2 standard deviations above and below the mean to identify observations that deviate substantially from the mean. Observations at these time points might indicate aberrant conditions, e.g., due to disturbance or even sample contamination. Spaghetti plots, illustrating the longitudinal trajectory of each individual, support visual assessment of individual subjects’ stability, identifying aberrant individuals and time points. Volatility charts, which combine the attributes of control charts and spaghetti plots, can be generated by using q2-longitudinal’s “volatility” visualizer (Fig. 1). This produces an interactive HTML-based visualization, allowing users to select which metric is plotted on the *y* axis, select which categorical sample metadata column is used to group (aggregate) individual samples into group averages at each time point, adjust color scheme and other plot formatting characteristics, and toggle error bars, control/warning limits, and “spaghetti” lines. See https://github.com/caporaso-lab/longitudinal-notebooks for a gallery of examples.

### Feature volatility.

A principal goal in longitudinal experiments is to determine how microbial communities (e.g., taxa, sequence variants, or other “features”) change over time. This may be, for example, in response to a treatment or during different stages of host development. The most common rudimentary way to do this is to simply examine the average relative compositions of species over time, e.g., as a bar plot or heatmap. In doing so, we see the most dominant taxa and their succession over time. This approach, however, is effective only for identifying the most abundant organisms, and longitudinal averages smooth the data, ignoring whether these features are actually associated with specific time points across individual subjects. Other approaches, such as looking at the magnitude of change in abundance between time points, are similarly blind to the temporal dependence of these organisms.

We implement a different approach in q2-longitudinal’s “feature-volatility” action, which uses machine learning regressors (random forests [13] by default) to learn the structure of the data and identify features (including low-abundance features) that are predictive of different states. Important features identified by these methods change over time, and their abundance is predictive of the specific time point when a sample is collected, indicating a temporal relationship. Importantly, feature importance does not imply statistical significance; this is intended as an exploratory method for identifying potentially relevant features for subsequent investigation, as illustrated below. The accuracy of the model itself can be assessed to determine how well these features differentiate time points. Only features from accurate models are likely to be temporally informative.

**FIG 2:**
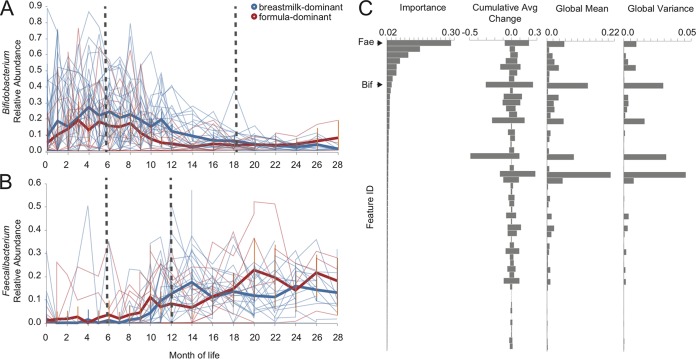
Longitudinal feature-volatility analysis of bacterial genera in the ECAM subjects. Relative abundances of *Bifidobacterium* (A) and *Faecalibacterium* (B) across time are shown both for individual subjects (narrow lines) and group averages (thick lines) categorized by predominant diet type (predominantly breastfed or formula fed during the first 3 months of life). Dashed lines indicate the developmental “windows” that were separately analyzed by LME as described in the text. C, feature metadata and other descriptive statistics for the top important features, ordered by decreasing importance. *Bifidobacterium* and *Faecalibacterium* are labeled “Bif” and “Fae,” respectively. This list was truncated and does not contain all 71 important genera identified in this analysis.

The feature-volatility action produces an interactive visualization of the longitudinal abundance, importance, and descriptive statistics for all important features (Fig. 2). The longitudinal abundance of each feature is plotted using volatility plots (as described above). In addition, feature importances, descriptive statistics, and other feature metadata are plotted as bar charts, guiding exploration of these features by comparing multiple feature characteristics. Users can select which features are plotted in the control charts either by using the “metric column” selection menu in the tool panel to the right of the plots or by clicking on the bar associated with that feature in the feature metadata bar plots. See https://github.com/caporaso-lab/longitudinal-notebooks for a gallery of examples.

### Longitudinal analysis of early-life microbiome development.

To demonstrate some of the methods currently available in q2-longitudinal, we present a reanalysis of data from the early childhood and the microbiome (ECAM) study (1). This study tracked the 16S rRNA gene microbiota compositions of 43 infants in the United States sampled at regular intervals from birth to 2 years of age and associations between antibiotic use, delivery mode, and predominant diet on microbiota composition and development. Here, we focus on novel methods implemented in q2-longitudinal, as well as other methods in q2-longitudinal, that draw new discoveries from the ECAM study data. Tutorials demonstrating all methods available in q2-longitudinal are available at https://github.com/qiime2/q2-longitudinal.

### Identification of longitudinally volatile features.

The original ECAM study focused primarily on particular taxa that were dominant in the childhood gut environment and were strongly associated with cesarean section (e.g., *Bacteroides*) and antibiotic use. Less attention was given to taxa associated with development of the childhood gut in general and to changes associated with early diet. To identify bacterial genera associated with early-life gut development and dietary modes, we applied q2-longitudinal’s feature-volatility action to the ECAM data set, showing that only a few genera constitute the most important features and can accurately predict a subject’s age (mean square error = 11.78, *R*^2^ = 0.79) using a holdout set for model validation; the top 10 features comprise 75.3% of the total feature importance (from among 71 total important features used to train the final model) (Fig. 2). From among these, we focused on two genera to examine their abundance in relation to host age and diet: *Bifidobacterium* (Fig. 2A) and *Faecalibacterium* (Fig. 2B). These genera were chosen based on their relatively high importance, mean abundance, and cumulative average change (Fig. 2C). *Bifidobacterium* exhibited a period of increase in relative frequency from 0 to 6 months of life in all subjects and then decreased from 6 to 18 months of life (Fig. 2A). *Faecalibacterium* exhibited a stable, low average frequency during the first 6 months of life and then increased from 6 to 12 months in most subjects (Fig. 2B).

We used LME models via q2-longitudinal’s “linear-mixed-effects” action to test whether the relative abundances of these genera were impacted by time (age) and subject characteristics (see Materials and Methods for more information on LME). We fit three separate linear models to examine *Bifidobacterium* abundance between 0 to 6 and 6 to 18 months of life and *Faecalibacterium* between 6 to 12 months of life, since the trajectories appear approximately linear within each of these biologically sensible developmental phases (Fig. 2). Month, delivery mode, diet (predominantly breastfed or formula fed for the first 3 months of life), and sex were used as fixed effects; random intercepts (subject identifier [ID]) and slopes (month of life) were applied as random effects. *Bifidobacterium* relative abundance at 6 months of life was significantly impacted by diet (*P* = 0.009), indicating higher relative abundance in dominantly breastfed children (by a factor of 0.33) (Table 1). A significant interaction was also observed between diet and delivery mode (*P* = 0.009) on *Bifidobacterium* relative abundance at 6 months of life (Table 1). However, no factors other than age (*P* < 0.001) significantly impacted *Bifidobacterium* relative abundance during months 0 to 6 (Table 2). *Faecalibacterium* relative abundance was significantly associated with the interaction of age and delivery mode (*P* = 0.021), indicating that monthly growth in *Faecalibacterium* relative abundance was reduced by a factor of 0.014 in children delivered by cesarean section (Table 3). However, no other factor significantly impacted *Faecalibacterium* relative abundance, though diet exhibited a nearly significant effect at baseline (*P* = 0.053) (Table 3).

**TABLE 1:** Linear mixed-effects model results for *Bifidobacterium* relative abundances between 6 and 18 months of life in the ECAM study^*a*^

Model	Variable or parameter	Estimate	SE	Z-score	*P* value
Fixed effects	(Intercept)	0.465	0.101	4.579	<0.001
	Delivery [T.vaginal]	–0.114	0.097	–1.181	0.238
	Diet [T.formula-dominant]	–0.33	0.126	–2.612	0.009
	Sex [T.male]	–0.064	0.086	–0.743	0.457
	Delivery [T.vaginal]:diet [T.fd]	0.46	0.177	2.605	0.009
	Month	–0.015	0.017	–0.904	0.366
Random effects	Intercept (subject ID)	0.037	0.132		
	Slope (change per mo)	0.012	0.02		
	Covariance (intercept, time)	–0.004	0.228		

a Parameter estimate (coefficient), standard error, Z score, and *P* value for each model parameter. Brackets indicate reference groups for interpreting fixed-effect estimates.

**TABLE 2:** Linear mixed-effects model results for *Bifidobacterium* relative abundances between 0 and 6 months of life in the ECAM study^*a*^

Model	Variable or parameter	Estimate	SE	Z-score	*P* value
Fixed effects	(Intercept)	0.113	0.037	3.025	0.002
	Delivery [T.vaginal]	0.058	0.033	1.735	0.083
	Diet [T.formula-dominant]	–0.056	0.037	–1.486	0.137
	Sex [T.male]	–0.029	0.036	–0.827	0.408
	Month	0.022	0.006	3.552	<0.001
Random effects	Intercept (subject ID)	0.006	0.019		
	Slope (change per mo)	0.001	0.002		
Residual error	Covariance (intercept, time)	–0.001	0.005		

a Parameter estimate (coefficient), standard error, Z score, and *P* value for each model parameter.

**TABLE 3:** Linear mixed-effects model results for *Faecalibacterium* relative abundances between 6 and 12 months of life in the ECAM study^*a*^

Model	Variable or parameter	Estimate	SE	Z-score	*P* value
Fixed effects	(Intercept)	–0.049	0.039	–1.271	0.204
	Delivery [T.vaginal]	–0.087	0.048	–1.827	0.068
	Diet [T.formula-dominant]	0.028	0.014	1.939	0.053
	Sex [T.male]	–0.011	0.014	–0.781	0.435
	Month	0.008	0.005	1.646	0.1
	Month:delivery [T.vaginal]	0.014	0.006	2.302	0.021
Random effects	Intercept (subject ID)	0.008	0.082		
	Slope (change per mo)	0.0	0.001		
Residual error	Covariance (intercept, time)	–0.001	0.01		

a Parameter estimate (coefficient), standard error, Z score, and *P* value for each model parameter.

### Tracking temporal changes in subjects’ beta diversities.

Next, we sought to explore how beta diversity changed across time within and between groups in the ECAM study. q2-longitudinal contains multiple methods for visualizing and transforming longitudinal data, allowing us to examine individual trajectories in detail. In particular, first differences and first distances (see Materials and Methods for more details) enable inspection of individuals’ rates of incremental change between time points, an analysis not considered in the original ECAM study.

**FIG 3:**
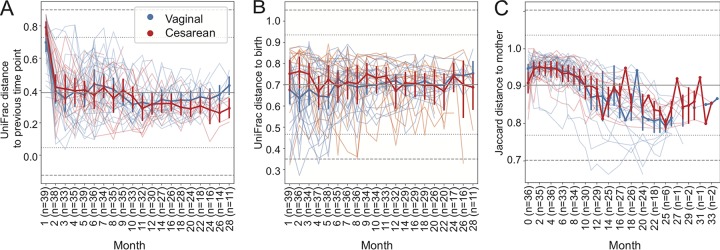
Volatility charts of longitudinal change in unweighted UniFrac distances between successive samples collected from the same subject (first distances) in the ECAM data set (A), distance from baseline for each subject (B), and Jaccard distance (proportion of features not shared) between children’s and their mothers’ stool microbiotas. Thick lines with error bars represent mean distance (± standard deviation) for vaginally and cesarean section-delivered subjects. Faded spaghetti lines represent the longitudinal trajectory for each individual subject. Horizontal lines represent the mean (solid midpoint) and 2 (dotted line) and 3 (dashed line) standard deviations from the mean computed across all samples. Sample sizes differ between subplots because some subjects are missing samples for a particular month, resulting in fewer subjects eligible for first differencing at that month and the subsequent time point. Note that *x* and *y* axis scales differ across the three plots to highlight difference in the scale that is most informative for each analysis.

We applied first distances to examine how beta diversity (unweighted UniFrac distance [14] between successive samples collected from the same subject) changed over time in each subject in the ECAM study (Fig. 3). Results indicate that vaginally and cesarean section-delivered infants exhibit similar rates of phylogenetic transition (Fig. 3A). This was marked by a dramatic shift in the first month of life, followed by gradual stabilization in the rate of change but a very large degree of variance. These groups diverged only in the first month of life (when cesarean section-born infants exhibited a higher degree of change within individuals) and after 2 years of life (when sample sizes and statistical power were lower). Consistently with the close similarity between delivery modes, an LME test indicates no significant differences between delivery modes, diet, or sex (data not shown).

The “first-distances” method also has a “baseline” parameter for calculating distance from a static time point (Fig. 3B). This can be a useful approach for assessing how a subject differs from the start/end of a study or from another static time point (e.g., to highlight fluctuations in community structure/composition related to a treatment) (Fig. 3B). This method accentuates the differences between vaginally and cesarean section-delivered infants during the first few months of life: cesarean section-delivered children exhibit greater phylogenetic change from baseline than do vaginally delivered children (Fig. 3B). Nevertheless, LME models indicate no significant differences between delivery modes, diet, or sex during this time period (testing months 0 to 6) or across the entire study period (2 separate tests [data not shown]).

### Quantifying shared features across time.

The first-distances method also allows us to track longitudinal change in the proportion of features that are shared between an individual’s samples. This can be performed by calculating pairwise Jaccard distance (the proportion of features that are not shared) between each pair of samples with QIIME 2’s “diversity” plugin and by using the “first-distances” method to extract distances between successive samples or from baseline. Furthermore, the baseline parameter in the first distances and first differences methods also provides the ability to track longitudinal change from a separate set of (nonlongitudinal) samples that are linked to those samples. The ability to compare longitudinal samples to a set of static reference samples supports many different types of questions pertinent to longitudinal microbiome experiments, e.g., comparing similarity between the microbiotas of human patients or gnotobiotic animals receiving fecal microbiota therapy and the compositions of donor samples (7), between fermentations and their inocula, or between intact and disturbed environments during recovery from disturbance.

We used first distances to track the number of shared features (Jaccard distance) between the stool microbiotas of infants and the stool microbiotas of their mothers near the time of birth in the ECAM data set (Fig. 3C). Jaccard distance between sequence variant profiles indicates that very few variants were shared with a child’s mother during the first year of life, but distance decreases into the second year of life, when a higher proportion of sequence variants were shared between mother-infant dyads (Fig. 3C). A LME test indicates a significant impact from diet (*P* = 0.001) and month of life (*P* < 0.001) on baseline Jaccard distance (Table 4). These results indicate that infants are born with various levels of similarity to their mothers and that initial dietary inputs alter this; formula feeding reduces baseline dissimilarity. As infants age, they accumulate more microbiota characteristics of an adult gut ecosystem, causing their gut microbiota to more closely resemble that of their mothers.

**TABLE 4:** Linear mixed-effects model results for Jaccard distances between stool bacterial compositions of infants between 0 and 34 months of age and their mothers near the time of birth^*a*^

Model	Variable or parameter	Estimate	SE	Z-score	*P* value
Fixed effects	(Intercept)	0.97	0.012	83.095	<0.001
	Delivery [T.vaginal]	–0.004	0.012	–0.383	0.702
	Diet [T.formula-dominant]	–0.04	0.012	–3.458	0.001
	Sex [T.male]	0.009	0.011	0.833	0.405
	Month	–0.006	0	–12.063	<0.001
Random effects	Intercept (subject ID)	0.001	0.007		
	Slope (change per mo)	0.0	0.0		
Residual error	Covariance (intercept, time)	0.0	0.0		

a Parameter estimate (coefficient), standard error, Z score, and *P* value for each model parameter.

### Conclusions.

Longitudinal designs for microbiome studies provide valuable information about temporal trends in biological activity. In addition, these designs allow investigators to distinguish between within- and between-subject variation, an important issue in characterizing heterogeneity in temporal patterns across experiments.

The q2-longitudinal plugin supports a variety of paired-sample and longitudinal tests relevant to studies of host-associated and environmental microbiomes. This includes methods for paired difference and distance testing, LME, microbial interdependence, analyses of volatility, and a variety of functions for generating interactive plots for data exploration and publication. Additional functions will be added to this plugin as they are developed (e.g., additional methods for quantifying longitudinal volatility and shared species counts), and we welcome collaboration from other developers who would like their methods accessible through q2-longitudinal (get in touch on the QIIME 2 Forum at https://forum.qiime2.org/). This plugin is included in QIIME 2, and installation instructions and tutorials can be accessed at https://qiime2.org or https://github.com/qiime2/q2-longitudinal.

## MATERIALS AND METHODS

The q2-longitudinal package (https://github.com/qiime2/q2-longitudinal) is written in Python 3 and is accessible as a QIIME 2 plugin (https://qiime2.org). As a plugin for QIIME 2, users automatically have access to q2-longitudinal simply by installing QIIME 2 and can interact with the plugin using a variety of user interfaces (command line, Python API, and graphical user interfaces are included in the Core Distribution). The actions in this plugin utilize SciPy (https://scipy.org), NumPy (15), and pandas (16) for data manipulation and statistical testing, q2-sample-classifier (17) for supervised regression, Vega (18) for interactive HTML-based visualizations, and Matplotlib (19) and seaborn (https://zenodo.org/record/12710) for static plots. Tutorials and other information about the q2-longitudinal plugin are available at https://github.com/qiime2/q2-longitudinal. This package is released under a 3-clause BSD license and is freely available, including for commercial use.

We make extensive use of LME in this work and so will describe this method and some data transformations in more detail here.

### Feature-volatility action.

The feature-volatility action uses a supervised learning regressor to predict a continuous variable (e.g., age or time) as a function of feature composition (e.g., taxonomic composition). q2-longitudinal wraps q2-sample-classifier (17) to access multiple different scikit-learn (20) supervised learning regressors (random forests [13] by default).

1. Samples are randomly split into training and testing sets (4:1 ratio).
2. The training set is used to train a user-selected regressor.
  - If “parameter-tuning” is true, cross-validated hyperparameter autotuning will be performed on the training set to select samples that optimize predictive accuracy. See q2-sample-classifier (17) for more details. If “parameter-tuning” is false, default parameters will be used; see scikit-learn (20) for details on default parameters for each regressor.
  - Cross-validated recursive feature elimination is performed on the training set to select features that optimize predictive accuracy and eliminate noisy features. See q2-sample-classifier (17) for more details.
  - Feature importance is extracted from the regression model. See scikit-learn (20) for details on each regressor.
3. The accuracy of the final optimized, trained model is determined by predicting values for the test set.
4. Interactive feature volatility plots are generated using Vega (18). These contain volatility plots of longitudinal abundance for each important feature, accompanied by bar charts comparing descriptive statistics (mean, median, variance, standard deviation, coefficient of variation, net average change, cumulate positive/negative change) for all features included in the final model.

### Linear mixed effects.

LME models examine the relationship between one or more independent variables (effects) and a single longitudinal response, where observations are made across dependent samples, e.g., in repeated-measures experiments. For example, a simple LME model may include an intercept and slope term as both fixed and random effects. The fixed intercept and slope can be interpreted as the regression line for the average subject, while the random effects reflect individual departures from the average line for each subject. This linear model can be written as *y_ij_* = *X′_ij_β + Z_ij_b_i_ + ε_ij_*, where *y_ij_* is the *j*th measurement on the *i*th participant; *X_ij_* is a *P × *1 vector of fixed-effect covariates that may include, e.g., time, group, gender, and their interactions; *β* is a *P × *1 vector of fixed-effect regression coefficients; *Z_ij_* is a *q *×* *1 vector of fixed effects that typically includes a polynomial function of time; *b_i_* is a *q *×* *1 vector of random effects that reflect individual departures from the overall population average effects (*b_i_* is assumed to be multivariate normal, with a mean vector *o* and variance Σ, where Σ is a *q *by* q* matrix reflecting the covariances between the random effects); and ε*_ij_* are normally distributed error terms that have mean *o* and variance σ^2^ and are assumed independent across individuals and time.

An attractive feature of the model is that it allows the investigator to explicitly model heterogeneity in the initial value and slope across subjects. This is important for longitudinal microbiome studies where we expect heterogeneity in the temporal pattern across individuals. Fixed effects are factors that may reflect group or time and assess the overall effect of the factor on the response. Random effects reflect variation in these effects across subjects. LME models are available in the linear-mixed-effects action in q2-longitudinal. All LME models described in this work for analysis of the ECAM data set used month of life, diet, delivery mode, and sex as fixed effects and random intercepts (subject ID) and slopes (month of life) as random effects. Initially, LME models were fit with all interactions between the main effects as fixed effects, and then insignificant terms were removed from the model to focus on main effects and significant interactions in the final model. Tables 1 to 4 list all factors used as fixed effects in the final models.

The linear-mixed-effects action in q2-longitudinal uses statsmodels’ “mixedlm” function to compute LME models (21). This function computes *P* values based on each variable’s Z-score estimate with respect to the standard normal distribution (two-tailed, alpha = 0.05).

### First differences/distances.

Another way to view time series data is by assessing how the rate of change differs over time. We can do this through calculating first differences, which represent the magnitude of change between successive time points for a given metric. If *Y_t_* is the value of single metric *Y* at time *t*, the first difference at time t is Δ*Y_t_* = *Y_t_* – *Y_t_*_– 1_. This calculation is performed at fixed intervals, so for each interval, Δ*Y_t_* is not calculated for subjects that are missing samples at time *t* or *t* – 1.

This transformation is performed in the “first-differences” method in q2-longitudinal. A similar method implemented in q2-longitudinal is first-distances, which instead identifies the beta diversity (between-sample) distances between successive samples from the same subject based on a distance matrix. The output of first-distances is particularly empowering, because it allows us to analyze longitudinal changes in beta diversity using actions that cannot operate directly on a distance matrix, such as linear mixed-effect models, or plotting with volatility charts.

### Difference/distance from baseline.

The first-differences and first-distances methods have an optional baseline parameter to instead calculate differences/distances from a static point (e.g., baseline or a time point when a treatment is administered): Δ*Y_t_* = *Y_t_* − *Y*_baseline_.

Calculating baseline differences/distances can help tease apart noisy longitudinal data to reveal underlying trends in individual subjects or highlight significant experimental factors related to changes in diversity or other dependent variables. This baseline can theoretically also come from a separate subject or set of reference samples. For example, we use first-distances in the results section to compare children’s stool microbiotas to their mothers’ stool microbiotas; see the example notebooks (https://github.com/caporaso-lab/longitudinal-notebooks) for more details on usage.

### Test data.

We use study data from the ECAM study (1) to demonstrate the features of q2-longitudinal. Raw sequence data (study ID 10249) were downloaded from Qiita (http://qiita.microbio.me) and analyzed with QIIME 2. Raw sequences were quality filtered using DADA2 (22) to remove phiX, chimeric, and erroneous reads. Sequence variants were aligned using MAFFT (23) and used to construct a phylogenetic tree using FastTree 2 (24). Beta diversity was estimated using unweighted UniFrac distance (14). All other analyses were performed using q2-longitudinal.

### Data availability.

Analysis data and notebooks used to generate all results in this study are available at https://github.com/caporaso-lab/longitudinal-notebooks.
